# Epidemiology and impact of device-specific infections on patients receiving left ventricular assist devices

**DOI:** 10.1016/j.jhlto.2025.100208

**Published:** 2025-01-17

**Authors:** Amit Iyengar, Jason Feinman, Joyce Jiang, Cindy Song, Spencer Kim, Alvin Mathew, Sophia Golec, Aarti Rao, Ankitha Radakrishnan, Michaela Asher, David Rekhtman, John DePaolo, Noah Moss, Shinobu Itagaki, Anelechi Anyanwu, Joyce Wald, Marisa Cevasco, Aditya Parikh

**Affiliations:** a Division of Cardiovascular Surgery, Hospital of the University of Pennsylvania, Philadelphia, Pennsylvania; b Division of Cardiovascular Medicine, Mount Sinai Health Systems, New York, New York; c Perelman School of Medicine, University of Pennsylvania, Philadelphia, Pennsylvania; d Division of Cardiovascular Medicine, Hospital of the University of Pennsylvania, Philadelphia, Pennsylvania; e Division of General Surgery, Hospital of the University of Pennsylvania, Philadelphia, Pennsylvania; f Division of Cardiovascular Surgery, Mount Sinai Health Systems, New York, New York

**Keywords:** left ventricular assist device, driveline infection, infectious disease, epidemiology, cardiac transplant

## Abstract

**Background:**

Left ventricular assist device-specific infections (LSIs) remain a persistent problem facing patients with durable left-ventricular assist devices (LVADs). However, the infectious agents and associated morbidity remained poorly defined. We sought to evaluate the incidence, epidemiology, and morbidity associated with LSIs in patients receiving modern centrifugal LVADs at 2 tertiary care centers.

**Methods:**

Retrospective analysis was performed of adult patients receiving HeartMate 3 implants at the University of Pennsylvania and Mount Sinai Health Systems from January 1, 2015 to March 31, 2021, with follow-up until March 31, 2022. Patients were grouped by history of LSI, defined as culture-positive infections and/or those requiring medical or surgical intervention. Demographic data, available culture data, medical interventions, and surgical interventions were queried. Survival analysis was censored at 4 years and landmarked according to 25th percentile time-to-infection.

**Results:**

Among 206 LVAD recipients, 71 (34.5%) developed an LSI. Predominant organisms were *Staphylococcus* (47.9%), *Pseudomonas* (15.5%), and *Serratia* (8.5%). Predictors of infection included Black race (LSI vs No LSI: 46.2% vs 29.2%, *p* = 0.021) and body mass index (median 29.7 vs 26.2 kg/m^2^, *p* = 0.007). Median time to infection was 231 days (112−423), with 19 (26.8%) patients requiring surgical debridement. Landmarked survival did not differ (log-rank *p* = 0.830). LSI patients were hospitalized an extra 8 (0−28) days for infection-related reasons.

**Conclusion:**

LSIs remain pervasive, with most related to *Staphylococcus*, *Pseudomonas*, and *Serratia*, and are associated with significantly increased rehospitalization burden. Surgical interventions were utilized in 26.8% of patients. Continued efforts to understand and prevent LSIs are necessary to improve care for LVAD patients.

Left ventricular assist devices (LVADs) are an increasingly used strategy for management of end-stage heart failure as both a bridge to transplantation and destination therapy.^1^ Over the past 2 decades, technological advancements have improved survival, device durability, hemocompatibility-associated complications, and patient quality of life.^2^, ^3^ However, infection remains a persistent problem, occurring in 18–59% of patients supported on durable LVADs.^2^ An International Society for Heart and Lung Transplantation (ISHLT) consensus conference defined 3 standardized categories of infections in LVAD patients: (1) LVAD-specific infections (LSIs), which are directly related to the device, occurring in the driveline, pump pocket, or internal surfaces (pump or cannula); (2) LVAD-related infections, which do not directly involve the device itself but may be a consequence of implantation; and (3) non-LVAD related infections.^4^, ^5^ With the growing duration of LVAD support and use as destination therapy in the modern heart failure landscape, LSIs are becoming increasingly prevalent.^2^ However, the causative infectious agents, morbidity, and resource utilization burden of LSIs remain poorly defined, particularly in current generations of devices. Thus, we sought to evaluate the incidence, epidemiology, and morbidity associated with LSIs in patients implanted with modern centrifugal left ventricular assist devices across 2 tertiary care LVAD centers.

## Methods

We performed a retrospective cohort study of all patients who underwent primary implantation of the HeartMate 3 (Abbott, Abbott Park, IL) durable LVAD between August 1st, 2015 and March 31st, 2021 at 2 academic, urban, tertiary referral hospitals—the University of Pennsylvania and Mount Sinai Health Systems. Patients were excluded from further analysis if their HeartMate 3 LVAD was placed for exchange of a previously implanted durable LVAD. This study was reviewed and approved by institutional review boards at the University of Pennsylvania and at Mount Sinai Health Systems. A data transfer agreement was put in place in accordance with protocols from both institutions. This study was performed in compliance with the ISHLT Ethics statement. The electronic medical record was queried for demographic information, preoperative risk profile, operative characteristics, and postoperative outcomes, as well as culture data and infection treatment if applicable. Follow-up data was collected continuously and censored on March 31st, 2022. On an outpatient basis, LVAD patients were surveilled a minimum of monthly by a multidisciplinary LVAD care team until they either underwent heart transplant or died. During this time, quality of life and LVAD performance, including driveline site examination, were closely monitored. If infection was suspected, further testing, including wound culture and blood culture, was performed to identify causative agent.

LSI was defined as driveline infection, pocket infection, or device infection that was culture-positive and/or required medical or surgical intervention.^4^ Patients were stratified into 2 groups based on whether they developed an LSI during the follow-up period. Descriptive statistics for continuous data were presented as medians with interquartile ranges for nonparametrically distributed variables or as means with standard deviations for parametrically distributed variables. Descriptive statistics for categorical data were presented as frequencies with percentages. Groups were compared using Kruskal-Wallis tests or student’s *t*-tests for continuous variables, and either χ2 or Fisher’s exact tests for categorical variables, where appropriate. The Shapiro-Wilk test was used to determine parametricity.

Outcomes of the study included survival after LVAD implantation, rehospitalization, surgical intervention for LSI including debridement or device exchange, and rate of heart transplant during the follow-up period. Cox proportional-hazards models for LSI development at 2 years post-HeartMate 3 implantation were developed. Candidate variables included pre-operative demographics, clinical characteristics, and surgical approach. Time-to-event analyses were performed utilizing Kaplan-Meier estimation, censored at 4 years post-operation. Landmarked Kaplan-Meier analyses, excluding patients who died within the 25th percentile for time to LSI, were performed to exclude cases of early mortality wherein patients did not survive long enough to develop an infection. Log-rank tests were used to determine statistical significance between groups. Analyses were performed using Stata/BE 17.0 (StataCorp, College Station, TX). Missingness of all but one variable (post-transplant length of stay) did not exceed 15%. Variables with missingness greater than 10% are reported in Table S1.

## Results

**Figure 1:**
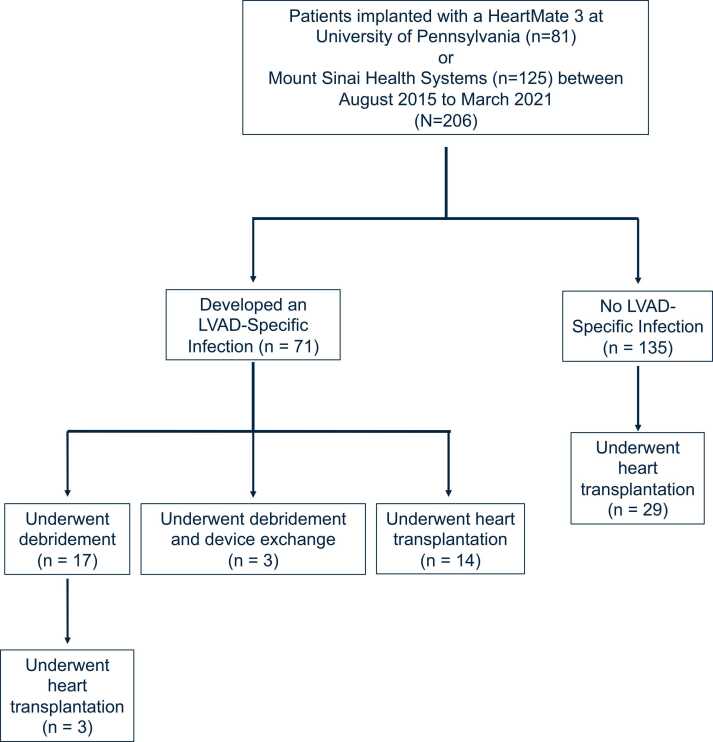
CONSORT diagram.

From August 2015 to March 2021, 206 patients were implanted with the HeartMate 3 at the 2 study centers, of which 71 (34.5%) developed an LSI (Figure 1). Baseline and operative characteristics are summarized in Table 1. LSI incidence was higher with Black race (46.2% vs 29.2%, *p* = 0.021) and elevated body mass index (29.7 vs 26.6 kg/m^2^, *p* = 0.007). Regression analysis further identified Black race as a risk factor for LSI development within 2 years of HeartMate 3 implantation (1.85 [1.04–3.28], *p* = 0.036, Table S2). Age, history of diabetes, cardiomyopathy type, INTERMACS (Interagency Registry for Mechanically Assisted Circulatory Support) score, pre-operative Impella or extracorporeal membrane oxygenation (ECMO) support, and surgical approach were similar across the 2 cohorts. Demographics stratified by center are shown in Table S3.

**Table 1:** Baseline and Operative Characteristics of Patients Receiving HeartMate 3, by Presence of LSI

Variable	Total (N = 206)	LSI (*n* = 71)	No LSI (*n* = 135)	*p* value
Demographics				
Age at surgery, years	60 (52−67)	60 (52−67)	59 (52−66)	0.527
Female sex	51 (24.8%)	22 (31.0%)	29 (21.5%)	0.133
Race				**0.039**
White	86 (46.5%)	21 (32.3%)	65 (54.2%)	**0.004**
Black	65 (35.1%)	30 (46.2%)	35 (29.2%)	**0.021**
Hispanic	24 (13.0%)	10 (15.4%)	14 (11.7%)	0.472
Asian	10 (5.4%)	4 (6.2%)	6 (5.0%)	0.491
BMI, kg/m^2^	27.3 (23.9−32.0)	29.7 (24.6−35.7)	26.6 (23.6−30.1)	**0.007**
BSA, m^2^	1.99 (1.83−2.19)	2.04 (1.84−2.26)	1.97 (1.82−2.12)	0.059
Diabetes	100 (48.5%)	35 (49.3%)	65 (48.2%)	0.876
ESRD on RRT	27 (13.2%)	4 (5.6%)	23 (17.2%)	**0.020**
CKD	120 (58.3%)	45 (63.4%)	75 (55.6%)	0.279
Hypertension	149 (72.3%)	53 (74.7%)	96 (71.1%)	0.590
Tobacco Use	132 (64.4%)	49 (69.0%)	83 (61.9%)	0.314
Last Lab Values				
eGFR				0.905
≥60	71 (34.5%)	25 (35.2%)	46 (34.1%)	
31−59	115 (55.8%)	40 (56.3%)	75 (55.6%)	
≤30	20 (9.7%)	6 (8.5%)	14 (10.4%)	
Albumin, g/dl	3.49 (2.89−4.09)	3.43 (2.85−4.01)	3.52 (2.89−4.14)	0.168
Medications				
Beta Blockers	130 (64.4%)	43 (61.4%)	87 (65.9%)	0.527
ACEi/ARB/ARNI	80 (39.0%)	26 (36.6%)	54 (40.3%)	0.607
Cardiac Status				
Cardiomyopathy				0.887
IHD	83 (40.3%)	29 (40.9%)	54 (40.0%)	
NIHD	121 (58.7%)	41 (57.8%)	80 (59.3%)	
INTERMACS				0.096
1	37 (18.0%)	9 (12.7%)	28 (20.7%)	
2	56 (27.2%)	16 (22.5%)	40 (29.6%)	
3	101 (49.0%)	39 (54.9%)	62 (45.9%)	
≥4	12 (5.8%)	7 (9.9%)	5 (3.7%)	
Intent				0.382
Bridge to transplant	55 (27.4%)	16 (22.9%)	39 (29.8%)	
Destination therapy	134 (66.6%)	51 (72.9%)	83 (63.3%)	
Bridge to decision	12 (6.0%)	3 (4.3%)	9 (6.9%)	
Other mechanical circulatory support devices				
Pre-op Impella	13 (6.3%)	3 (4.2%)	10 (7.4%)	0.372
Pre-op ECMO	20 (9.7%)	3 (4.2%)	17 (12.6%)	0.054
Pre-op IABP	36 (17.5%)	6 (8.5%)	30 (22.2%)	**0.013**
Surgical Approach				0.094
Full Sternotomy	159 (77.2%)	50 (70.4%)	109 (80.7%)	
Alternative Access	42 (22.8%)	21 (29.6%)	26 (19.3%)	

ACEi, angiotensin converting enzyme inhibitor; ARB, angiotensin receptor blocker; ARNI, angiotensin receptor-neprilysin inhibitor; BMI, body mass index; BSA, body surface area; CKD, chronic kidney disease; ECMO, extracorporeal membrane oxygenation; eGFR, estimated glomerular filtration rate; ESRD on RRT, end stage renal disease on renal replacement therapy; IABP, intra-aortic balloon pump; IHD, ischemic heart disease; INTERMACS, Interagency Registry for Mechanically Assisted Circulatory Support score; LSI, LVAD-specific infection; NIHD, non-ischemic heart disease.

Categorical data are expressed as *n* (%) and continuous data as medians (interquartile range) or means (standard deviations) where appropriate. Bold type denotes *p* < 0.05.

**Figure 2:**
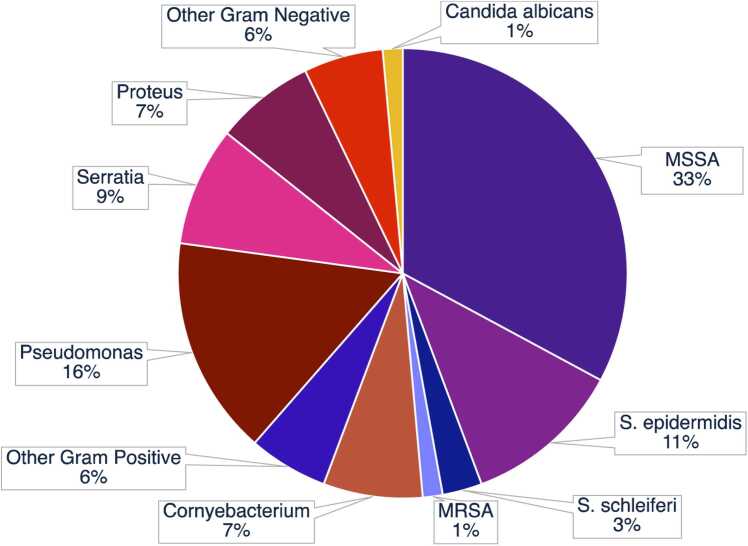
Culture data of LSIs. LSIs, left ventricular assist devices-specific infection; MRSA, methicillin-resistant *Staphylococcus aureus*; MSSA, methicillin-sensitive *Staphylococcus aureus.*

Characteristics of the 71 LVAD-specific infections are shown in Table 2 and Figure 2. The median time from device implant to LSI was 231 days. The majority were single organism infections, with rare polymicrobial (*n* = 5, 7.0%) and culture-negative (*n* = 4, 5.6%) cases. Complications of LSI included bloodstream infection (*n* = 15, 21.1%), mediastinitis (*n* = 9, 12.7%), and pocket infection (*n* = 12, 16.9%). Culture data showed that gram-positive bacteria (60.6%) were the predominant group. The most common genus of bacteria was *Staphylococcus* (47.9%); methicillin-sensitive *Staphylococcus aureus* (MSSA) (32.4%) and *S. epidermidis* (11.3%) were the most common species. *Corynebacterium* (7.0%) was the second most common gram-positive genera. Around 39.4% of LSIs were caused by gram-negative bacteria, of which *Pseudomonas* (15.5%), *Serratia* (8.5%), and *Proteus* (7.0%) were the most common genera. Fungal infection was seen only in 1 patient (1.4%). The median length of antimicrobial therapy was 361 days, with 77.5% requiring chronic suppressive treatment. Surgical interventions were needed in 19 cases (26.8%), including surgical debridement (*n* = 19, 26.8%) and device exchanges (*n* = 3, 4.2%). Device exchange was performed due to persistent infection refractory to antibiotic treatment and surgical debridement. All 3 patients that underwent device exchange were on destination therapy. Around 74.7% of patients required hospital admission to manage their LSI, with a median length of rehospitalization of 8 days. A breakdown of microbiological data by center is shown in Table S4.

**Table 2:** Culture Data and Treatment of HeartMate 3 Patients that Developed LSIs

Variable	Incidence (*n* = 71)
Microbiology	
Culture Negative	4 (5.6%)
Polymicrobial	5 (7.0%)
Gram Positive	43 (60.6%)
*Staphylococcus*	34 (47.9%)
MSSA	23 (32.4%)
*S. epidermidis*	8 (11.3%)
MRSA	1 (1.4%)
*S. schleiferi*	2 (2.8%)
*Cornyebacterium*	5 (7.0%)
*C. jeikeium*	1 (1.4%)
*C. striatum*	2 (2.8%)
*Enterococcus faecalis*	2 (2.8%)
*Streptococcus dysgalactiae*	1 (1.4%)
*Granulicatella adiacens*	1 (1.4%)
Gram Negative	28 (39.4%)
*Pseudomonas*	11 (15.5%)
*P. aeruginosa*	7 (9.9%)
*P. fluorescens*	1 (1.4%)
*Serratia*	6 (8.5%)
*S. marcescens*	2 (2.8%)
*Proteus*	5 (7.0%)
*P. mirabilis*	4 (5.6%)
*P. vulgaris*	1 (1.4%)
*Enterobacter cloaecae*	2 (2.8%)
*Stenotrophomonas maltophilia*	1 (1.4%)
*Acinetobacter baumannii*	1 (1.4%)
Fungal	1 (1.4%)
*Candida albicans*	1 (1.4%)
Treatment Course	
Time to Infection, days	231 (112−423)
Bloodstream Infection	15 (21.1%)
Mediastinitis	9 (12.7%)
Pocket Infection	12 (16.9%)
Length of Antimicrobial Therapy, days	361 (141−604)
Required Suppressive Antimicrobial Therapy	55 (77.5%)
Required Surgical Reintervention	19 (26.8%)
Surgical Driveline Debridement	19 (26.8%)
Device Exchange	3 (4.2%)
Required Hospitalization for LSI	53 (74.7%)
Number of LSI-Related Hospitalizations	1 (0−2)
Days Hospitalized for LSI	8 (0−28)

LSI, LVAD-specific infection; LVAD, left ventricular assist device; MRSA, methicillin-resistant *Staphylococcus aureus;* MSSA, methicillin-sensitive *Staphylococcus aureus.*

Categorical data are expressed as *n* (%) and continuous data as medians (interquartile range).

**Figure 3:**
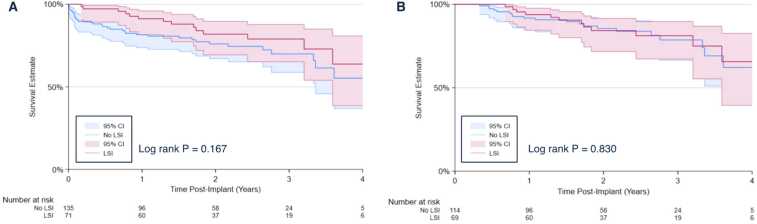
Kaplan Meier survival estimates at 4-years post-HeartMate 3 implantation, by presence of LSI. (A) Unadjusted survival estimates. Four-year survival estimates were 63.7% for the LSI cohort vs 55.2% for the no LSI cohort. (B) Patients who died in the first 112 days post-implant, corresponding to the 25th percentile for time to LVAD-Specific Infection, were censored from the analysis to exclude cases of early mortality wherein patients did not have sufficient time to develop an infection. Four-year survival estimates were 65.6% for the LSI cohort vs 62.2% for the no LSI cohort. CI, confidence interval; LSI, LVAD-specific infection; LVAD, left ventricular assist device.

Post-implant outcomes are shown in Table 3. Median follow-up time was 1.84 years. There was no association between length of stay of the index admission and LSI development (*p* > 0.05). The LSI cohort had a greater number (2 vs 1, *p* = 0.002) and length (12 vs 5 days, *p* = 0.002) of rehospitalizations at 1-year post-implant. Kaplan-Meier estimates of survival at 4 years post-LVAD were equivalent between patients who developed LSI and those who did not (63.7% vs 55.2%, log-rank *p* = 0.167, Figure 3A). When excluding patients with early mortality (within the first 112 days post-implant, corresponding to the 25th percentile for time to LSI) who may not have survived long enough to develop an infection, 4-year survival estimates still did not differ by presence of LSI (65.6% vs 62.2%, log-rank *p* = 0.830, Figure 3B).

**Table 3:** Post-Operative Outcomes of Patients Receiving HeartMate 3, by LSI

Variable	Total (N = 206)	LSI (*n* = 71)	No LSI (*n* = 135)	*p* value
In Hospital Outcomes				
Index Length of Stay, days	21 (17−34)	20 (14−32)	22 (18−35)	0.245
30-day Mortality	10 (4.9%)	0 (0%)	10 (7.4%)	**0.019**
Long Term Outcomes				
GI Bleed	69 (33.5%)	25 (35.2%)	44 (32.6%)	0.705
CVA	30 (14.6%)	12 (16.9%)	18 (13.3%)	0.490
Surgical Reintervention	21 (10.2%)	18 (25.4%)	3 (2.2%)	**<0.001**
Surgical Driveline Debridement	17 (8.3%)	17 (23.9%)	0 (0%)	**<0.001**
Pump Exchange	7 (3.4%)	4 (5.6%)	3 (2.2%)	0.187
Follow-up Time, days	671 (380−1,003)	735 (511−1,114)	618 (273−964)	**0.029**
Number of Rehospitalizations	2 (1−5)	4 (2−8)	1 (0−4)	**<0.001**
1-year post-implantation	1 (0−3)	2 (1−3)	1 (0−2)	**0.002**
Length of Rehospitalizations, days	21 (4−52)	39 (17−74)	10 (0−38)	**<0.001**
1-year post-implantation	6 (0−23)	12 (2−27)	5 (0−18)	**0.005**
Patient Status at Most Recent Follow-Up				
Mortality	54 (26.2%)	16 (22.5%)	38 (28.2%)	0.384
Transplant	46 (22.3%)	17 (23.9%)	29 (21.5%)	0.687
Alive w/Device	101 (49.0%)	33 (46.5%)	68 (50.4%)	0.595

CVA, cerebrovascular accident; GI, gastrointestinal; LSI, LVAD-specific infection; LVAD, left ventricular assist device.

Categorical data are expressed as *n* (%) and continuous data as medians (interquartile range). Bold type denotes *p* < 0.05.

**Figure 4:**
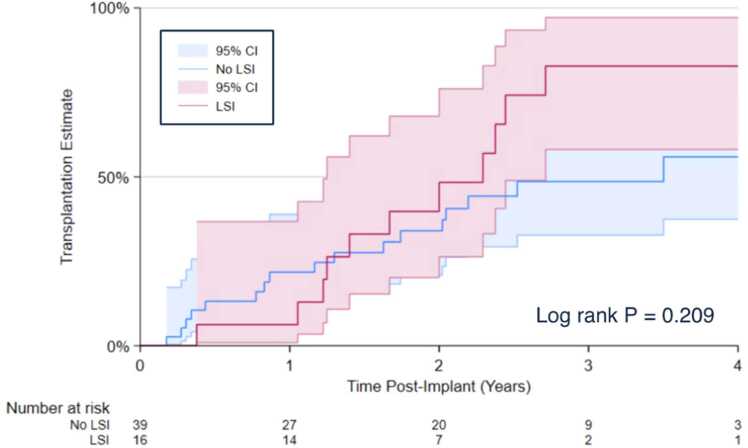
Kaplan-Meier estimates of rates of heart transplant at 4 years post-HeartMate 3 implantation in bridge to transplant patients, by presence of LSI. Four-year transplantation rates were 82.8% for the LSI cohort and 55.9% for the no LSI cohort. CI, confidence interval; LSI, LVAD-specific infection; LVAD, left ventricular assist device.

Forty-six patients ultimately underwent heart transplantation, of which 17 (37%) had a history of LSI. Post-transplant outcomes are summarized in Table 4. Median time to transplant was 572 days and did not vary by history of LSI. In patients intended for bridge-to-transplant use of the LVAD, there was no statistically significant difference in rate of transplant at 4 years after LVAD implantation by history of LSI (82.8% vs 55.9%, log-rank *p* = 0.209) (Figure 4). There was no difference in intensive care unit (ICU) length of stay, in-hospital death, or graft rejection by incidence of LSI (all *p* > 0.05).

**Table 4:** Post-Transplant Outcomes of HeartMate 3 Patients that Underwent Heart Transplantation, by LSI

Variable	Total (N = 46)	LSI (*n* = 17)	No LSI (*n* = 29)	*p* value
Time to Transplant, days	572 (274−774)	619 (385−867)	519 (265−724)	0.173
ICU Length of Stay, days	9 (6−15)	10 (7−11)	8 (6−18)	0.862
Length of Stay, days	24 (17−37)	24 (15−37)	27 (18−38)	0.408
In Hospital Death	6 (13.0%)	4 (23.5%)	2 (6.9%)	0.124
Follow-Up				
Rejection Within 1 Year	4 (9.8%)	1 (6.3%)	3 (12.0%)	0.488
Any Graft Rejection	5 (12.2%)	1 (6.3%)	5 (12.2%)	0.341
Mortality Within 1 Year	6 (17.7%)	4 (30.8%)	2 (9.5%)	0.133

ICU, intensive care unit; LSI, LVAD-specific infection; LVAD, left ventricular assist device.

Categorical data are expressed as *n* (%) and continuous data as medians (interquartile range). Bold type denotes *p* < 0.05.

## Discussion

Device-specific infection remains pervasive in modern LVAD patient cohorts.^3^, ^6^, ^7^, ^8^ We report a 35% prevalence of LSI in HeartMate 3 patients across 2 tertiary care LVAD centers. Most infections were related to *Staphylococcus, Pseudomonas,* and *Serratia,* and 28.2% required surgical intervention*.* While development of LSI did not impact 4-year survival or time to transplantation, LSI was associated with significantly increased rehospitalization burden and surgical reintervention.

Although rates of thrombosis, stroke, bleeding events, pump exchange, mortality, and overall infection have decreased with the transition from pulsatile-flow to smaller continuous-flow devices that require less surgical time and extent of dissection,^3^, ^7^, ^9^ modern centrifugal-flow pumps (i.e. HeartMate 3), designed specifically to reduce hemocompatibility-related adverse events, have not been shown to offer any reduction of LSI risk, compared to their predecessor, axial-flow pumps.^1^, ^2^, ^6^ We report a 34.5% prevalence of LSI in patients receiving the HeartMate 3 between 2015 and 2021 with a median follow up time of 1.8 years, which is within previously reported ranges for durable LVAD models.^2^ Despite advancements in device design, increasing duration of LVAD support and more frequent use as destination therapy, the incidence^1^, ^2^, ^6^ and pathogens^4^ of infection in LSI have not changed over time. Few epidemiological reports of LSI are specific to the HeartMate 3 model, but the epidemiology of LSI in our cohort of patients is overall similar to that of past reports of LSI that include HeartMate 3, HeartMate 2, and HeartWare, with some minor differences at the genus level largely attributable to sample size. Overall, gram-positive bacteria were the most common group, followed by gram-negative bacteria and, rarely, fungi. There were no instances of mycobacterial LSI in our cohort, although these have been previously described.^2^, ^10^ Consistent with previous studies, *Staphylococcus spp.,* most frequently *S. aureus* and *S. epidermidis*,^2^, ^10^ which form biofilms that increase virulence, treatment course, and risk for bloodstream infection,^11^, ^12^ were responsible for nearly half of LSIs in our series (Table 2). Among gram-negative species, *Pseudomonas*, which similarly challenges treatment because of its capability to form biofilms, was the most common in our series, while we observed more cases of *Serratia* and *Proteus*, and fewer cases of *Klebsiella* and *Enterobacter* than other reports.^2^, ^10^ The continued prevalence of skin colonizers and biofilm-capable bacteria in LSI epidemiology demonstrates that the percutaneous driveline site remains an access point to infection, and that the velour driveline covering is susceptible to colonization. Although newer LVADs have smaller pump size and thinner, more flexible drivelines in an effort to decrease surface area and trauma-induced infection, LSI rates have not diminished.^7^, ^13^, ^14^ Efforts to develop fully internally powered LVADs that eliminate the need for drivelines will be critical in reducing the incidence and morbidity of LSIs.

In our series, LSI was associated with Black race (46.2% vs 29.2%, *p* = 0.021, Table 1) and higher BMI (29.7 vs 26.6 kg/m^2^, *p* = 0.007, Table 1). Regression analysis further identified Black race as a risk factor for LSI development within 2 years post-implantation (1.85 [1.04–3.28], *p* = 0.036, Table S2). There have been conflicting reports in the literature suggesting that obesity may increase infection risk. Increased adipose tissue may cause poor vascularization, impairing wound healing, and complicate aseptic wound care.^2^, ^15^, ^16^, ^17^ Obesity has been associated with immune dysregulation,^16^, ^17^ although interestingly diabetes, which is also previously reported as a risk factor for LSI by this mechanism,^2^, ^4^, ^8^, ^13^ was not associated with increased LSI in this study. Importantly, obesity is also associated with social determinants of health (SDOH) that lead to healthcare disparities.^18^, ^19^ Black race is similarly associated with greater SDOH burden,^18^ and has been shown to increase risk of cardiovascular disease,^20^ decrease access to LVAD treatment and heart transplant,^21^ and increase comorbidities that are risk factors for bacteremia.^22^ Since current strategies for LSI prevention focus on driveline maintenance and frequent monitoring to increase early diagnosis,^8^, ^23^ further addressing SDOH that limit access to healthcare may prove an actionable means to reduce LSI burden.

Management of LVAD-specific infection differs by institution, but inpatient admission is often required for further evaluation, intravenous antimicrobials, and sometimes surgical management.^8^ Currently, the ISHLT recommends antibiotic treatment for superficial LSI until the infection clears, and initial antibiotic treatment combined with surgical debridement, pump replacement, and/or suppressive antibiotic therapy as needed for deeper LSI (Class I, level of evidence C).^4^ However, the ISHLT does not make specific recommendations for choosing between driveline site debridement, pump replacement, and suppressive antibiotics.^4^ In our cohort, 74.7% of LSIs required hospitalization, and 26.8% required surgical intervention, including 19 driveline debridement cases and 3 device exchanges (Table 2). Although surgical intervention may be seen as the most definitive management, it comes with compounded morbidity and mortality risk—LVAD exchange in particular is associated with poor outcomes (41% survival at 1 year, and even poorer when performed for infection).^7^ A 2018 systematic review found that LVAD exchange had no clear benefit over non-exchange treatment modalities (including both medical management and other, less invasive, procedures).^24^ Furthermore, recurrence or progression of LSI can occur despite surgical intervention, reported in 54% of cases.^25^ Overall, extent and necessity of surgical management for LSI should be decided on an individual patient basis, with consideration for procedural morbidity and response (or lack thereof) to more conservative measures.

Given the frequent need for inpatient management, our observation that LSI was associated with rehospitalizations of higher frequency and duration (Table 4) is hardly surprising, and is consistent with the findings of Akhter et al., where pump-related infection accounted for 21% of readmissions.^26^ Hospitalization for infection has been reported to incrementally increase costs for LVAD patients by between $22,000 and $53,000^27^, ^28^ and negatively impact quality of life in LVAD patients through feelings of uselessness and lack of control, resulting in both an individual and systems-level burden.^29^, ^30^

In the REMATCH trial, despite the 48% reduction in all-cause mortality in the first year in the LVAD arm, 41% of deaths in the LVAD cohort were due to infection.^31^ Our data, with a median follow-up time of 1.8 years, did not show an increase in landmarked mortality in patients that developed an LSI (Figure 3), likely due to sample size. Given the perceived increased mortality risk, LSI has been suggested as an indication for urgent transplantation, rather than a contraindication,^24^, ^32^, ^33^, ^34^ as the severity of illness may preclude device removal and transplant can effectively treat the device infection. Under current heart allocation guidelines, bridge to transplant (BTT) patients with an LSI may increase in waitlist urgency from status 4 to status 3. However, it is certainly possible that serious infection could result in temporary inactivation and reclassification as status 7, delaying transplant. We did not observe a statistically significant association between history of LSI and delayed heart transplantation (Figure 4), increased 1-year post-transplant rejection, or increased mortality (Table 4), although the number of patients who underwent transplant in our series was low. Previous reports (also with low numbers) have similarly suggested that controlled LSI does not impact post-transplant outcomes and should thus not affect transplant candidacy.^35^ However, it has also been previously suggested that LSI can complicate post-transplant hemodynamics, contributing to post-bypass vasoplegia in a series by Parikh et al.^36^ In the current series, post-transplant in-hospital mortality was higher in the LSI group (23.5% vs 6.9%, *p* = 0.124) (Table 4), suggesting a signal for increased heart transplant morbidity which may be limited to the perioperative period.

Limitations to this study include all those associated with its retrospective design. Notably, our analysis does not include data surrounding the agents used for antimicrobial therapy. The epidemiological data regarding infection microbiology may also be more reflective of regional distributions. Additionally, differences between the 2 study centers in LVAD management and LSI surveillance, diagnosis, and treatment protocols may confound the results. Interpretation of findings is limited by the sample size.

Overall, our data shows that LSIs remain a pervasive issue facing modern LVAD recipients that holds a significant rehospitalization burden, resulting in greater resource utilization and negative impact on patient quality of life. LSI reduction must be the next focus of engineering efforts to improve LVAD care. As LVADs are increasingly used as destination therapy, the next focus must be on equitable access to regular, high-quality LVAD follow-up care for driveline maintenance and innovation of fully internal devices that eliminate the need for drivelines altogether.

## Disclosure statement

None of the authors of this paper have any financial conflicts of interest or sources of funding relevant to this research to report.

## Author contributions

A.I., J.F., J.J., and M.C. conceived the study design. Data was collected by J.F., J.J., S.K., A.M., S.G., A.R., A.R., and M.A. Data analysis and figures were made by J.J. and S.K. with significant support from A.I., J.F, M.A., and M.C. The manuscript was written by A.I., J.J., and C.S with guidance and contribution from J.F., A.M., S.G., A.R., A.R., D.R., J.D., N.M., S.I., A.A., J.W., M.C., and A.P.

## Footnotes

Appendix A Supplementary data associated with this article can be found in the online version at doi:10.1016/j.jhlto.2025.100208.
