# Supplementary material for: Epidemiology and impact of device-specific infections on patients receiving left ventricular assist devices
Source: JHLT Open. 2025 Jan 17;8:100208. doi: 10.1016/j.jhlto.2025.100208 (PMC11935351; doi:10.1016/j.jhlto.2025.100208)
Supplement: Supplementary file 1 — Supplementary material [file mmc1.docx]

**Table S1.** Variables with greater than 10% missingness.

| **Variable** | **Total**  **(N = 206)** | **LSI (n = 71)** | **No LSI (n = 135)** |
| --- | --- | --- | --- |
| ***Baseline demographics*** |  |  |  |
| **Race** | 10.2% | 8.5% | 11.1% |
|  |  |  |  |
| ***Post-transplant outcomes*** |  |  |  |
| **ICU Length of Stay, d** | 10.9% | 5.9% | 13.8% |
| **Length of Stay, d** | 15.2% | 5.9% | 20.7% |
| **In Hospital Death** | 13.0% | 23.5% | 6.9% |
| **Rejection Within 1 Year** | 10.9% | 5.9% | 13.8% |
| **Any Graft Rejection** | 10.9% | 5.9% | 13.8% |
| **Mortality Within 1 Year** | 10.9% | 5.9% | 13.8% |

LSI, LVAD-specific infection; ICU, intensive care unit.

**Table S2.** Cox proportional-hazards model for LSI development at 2-years post-HeartMate 3 implantation.

| **Variable** | **Hazard Ratio [95% CI]** | **P Value** |
| --- | --- | --- |
| **Demographics** |  |  |
| **Age, y (increasing)** | 1.00 [0.98-1.02] | 0.750 |
| **Female sex** | 1.02 [0.60-1.74] | 0.939 |
| **Race** |  |  |
| **Black vs White** | 1.85 [1.04-3.28] | **0.036** |
| **Hispanic vs White** | 1.55 [0.68-3.53] | 0.292 |
| **Asian vs White** | 1.93 [0.66-5.66] | 0.228 |
| **BMI, kg/m^2^ (increasing)** | 1.02 [0.99-1.06] | 0.183 |
| **BMI≥30, kg/m^2^** | 1.17 [0.72-1.90] | 0.516 |
| **BMI≥25, kg/m^2^** | 1.23 [0.69-2.20] | 0.473 |
| **BSA, m^2^ (increasing)** | 0.91 [0.37-2.25] | 0.838 |
| **Diabetes** | 0.81 [0.50-1.32] | 0.400 |
| **ESRD on RRT** | 0.44 [0.16-1.20] | 0.108 |
| **CKD** | 1.27 [0.77-2.09] | 0.343 |
| **Hypertension** | 1.01 [0.59-1.74] | 0.961 |
| **Tobacco Use** | 1.26 [0.74-2.13] | 0.392 |
| **Albumin, g/dL (increasing)** | 0.82 [0.56-1.20] | 0.313 |
|  |  |  |
| **Medications** |  |  |
| **Beta blockers** | 0.66 [0.40-1.09] | 0.107 |
| **ACEi/ARB/ARNI** | 0.89 [0.55-1.46] | 0.655 |
|  |  |  |
| **Cardiac Status** |  |  |
| **IHD vs NIHD** | 1.17 [0.71-1.91] | 0.540 |
| **INTERMACS** |  |  |
| **1 vs ≥4** | 0.23 [0.08-0.62] | **0.004** |
| **2 vs ≥4** | 0.21 [0.09-0.53] | **0.001** |
| **3 vs ≥4** | 0.23 [0.10-0.54] | **0.001** |
| **Intent** |  |  |
| **Destination therapy vs bridge to transplant** | 1.40 [0.78-2.50] | 0.259 |
| **Bridge to decision vs bridge to transplant** | 1.24 [0.36-4.28] | 0.737 |
| **Other mechanical circulatory support devices** |  |  |
| **Pre-op Impella** | 0.79 [0.25-2.52] | 0.689 |
| **Pre-op ECMO** | 0.58 [0.18-1.86] | 0.363 |
| **Pre-op IABP** | 0.54 [0.23-1.26] | 0.155 |
|  |  |  |
| **Surgical Approach** |  |  |
| **Alternative access vs full sternotomy** | 1.67 [1.00-2.81] | 0.052 |

Univariable hazard ratios for graft failure with 95% confidence interval (CI) and p-value for interaction were calculated for association. Bold type denotes P < 0.05. LSI, LVAD-specific infection; BMI, body mass index; BMI, body surface area; ESRD on RRT, end stage renal disease on renal replacement therapy; CKD, chronic kidney disease; eGFR, estimated glomerular filtration rate; ACEi, angiotensin converting enzyme inhibitor; ARB, angiotensin receptor blocker; ARNI, angiotensin receptor-neprilysin inhibitor; IHD, ischemic heart disease; NIHD, non-ischemic heart disease; INTERMACS (Interagency Registry for Mechanically Assisted Circulatory Support) score; ECMO, extracorporeal membrane oxygenation; IABP, intra-aortic balloon pump.

**Table S3.** Baseline and operative characteristics of patients receiving HeartMate 3, by presence center.

| **Variable** | **Total**  **(N = 206)** | **University of Pennsylvania (n = 81)** | **Mount Sinai Health Systems (n = 125)** | **P Value** |
| --- | --- | --- | --- | --- |
| **Demographics** |  |  |  |  |
| **Age at surgery, y** | 60 (52-67) | 50 (51-68) | 61 (53-67) | 0.493 |
| **Female sex** | 51 (24.8%) | 14 (17.3%) | 37 (29.6%) | **0.045** |
| **Race** |  |  |  | **0.001** |
| **White** | 86 (46.5%) | 47 (58.0%) | 39 (31.2%) | **<0.001** |
| **Black** | 65 (35.1%) | 22 (27.2%) | 43 (34.4%) | 0.172 |
| **Hispanic** | 24 (13.0%) | 4 (4.9%) | 20 (16.0%) | **0.011** |
| **Asian** | 10 (5.4%) | 2 (2.5%) | 8 (6.4%) | 0.152 |
| **BMI, kg/m^2^** | 27.3 (23.9-32.0) | 28.2 (24.9-33.1) | 26.4 (23.5-31.9) | 0.051 |
| **BSA, m^2^** | 1.99 (1.83-2.19) | 2.05 (1.89-2.23) | 1.96 (1.79-2.15) | **0.030** |
| **Risk factors** |  |  |  |  |
| **Diabetes** | 100 (48.5%) | 36 (44.4%) | 64 (51.2%) | 0.343 |
| **ESRD on RRT** | 27 (13.2%) | 22 (27.5%) | 5 (4.0%) | **<0.001** |
| **CKD** | 120 (58.3%) | 38 (46.9%) | 82 (65.6%) | **0.008** |
| **Hypertension** | 149 (72.3%) | 50 (61.7%) | 99 (79.2%) | **0.006** |
| **Tobacco Use** | 132 (64.4%) | 55 (68.8%) | 77 (61.6%) | 0.297 |
| **Last Lab Values** |  |  |  |  |
| **eGFR** |  |  |  | 0.128 |
| **≥ 60** | 71 (34.5%) | 32 (39.5%) | 39 (31.2%) |  |
| **31-59** | 115 (55.8%) | 45 (55.6%) | 70 (56.0%) |  |
| **≤ 30** | 20 (9.7%) | 4 (4.9%) | 16 (12.8%) |  |
| **Albumin, g/dL** | 3.49 (2.89-4.09) | 3.70 (3.11-4.28) | 3.35 (2.77-3.94) | **<0.001** |
| **Medications** | |  |  |  |
| **Beta Blockers** | 130 (64.4%) | 58 (74.4%) | 71 (58.1%) | **0.019** |
| **ACEi/ARB/ARNI** | 80 (39.0%) | 47 (58.0%) | 33 (26.6%) | **<0.001** |
|  |  |  |  |  |
| **Cardiac Status** |  |  |  |  |
| **Cardiomyopathy** | |  |  | 0.358 |
| **IHD** | 83 (40.3%) | 30 (37.0%) | 53 (42.4%) |  |
| **NIHD** | 121 (58.7%) | 51 (63.0%) | 70 (56.0%) |  |
| **INTERMACS** |  |  |  | **0.009** |
| **1** | 37 (18.0%) | 20 (24.7%) | 17 (13.6%) |  |
| **2** | 56 (27.2%) | 20 (24.7%) | 36 (28.8%) |  |
| **3** | 101 (49.0%) | 41 (50.6%) | 60 (48.0%) |  |
| **≥4** | 12 (5.8%) | 0 (0.0%) | 12 (9.6%) |  |
| **Intent** |  |  |  | **<0.001** |
| **Bridge to**  **transplant** | 55 (27.4%) | 19 (25.0%) | 36 (28.8%) |  |
| **Destination therapy** | 134 (66.6%) | 46 (60.5%) | 88 (70.4%) |  |
| **Bridge to decision** | 12 (6.0%) | 11 (14.5%) | 1 (0.8%) |  |
| **Other mechanical circulatory support devices** | | | | |
| **Pre-op Impella** | 13 (6.3%) | 11 (13.5%) | 2 (1.6%) | **0.001** |
| **Pre-op ECMO** | 20 (9.7%) | 16 (19.8%) | 4 (3.2%) | **<0.001** |
| **Pre-op IABP** | 36 (17.5%) | 18 (22.2%) | 18 (14.4%) | 0.149 |
|  |  |  |  |  |
| **Surgical Approach** |  |  |  | **0.001** |
| **Full Sternotomy** | 159 (77.2%) | 72 (88.9%) | 87 (69.6%) |  |
| **Alternative Access** | 42 (22.8%) | 9 (11.1%) | 38 (30.4%) |  |

Categorical data are expressed as n (%) and continuous data as medians (interquartile range) or means (standard deviations) where appropriate. Bold type denotes P < 0.05. LSI, LVAD-specific infection; BMI, body mass index; BMI, body surface area; ESRD on RRT, end stage renal disease on renal replacement therapy; CKD, chronic kidney disease; eGFR, estimated glomerular filtration rate; ACEi, angiotensin converting enzyme inhibitor; ARB, angiotensin receptor blocker; ARNI, angiotensin receptor-neprilysin inhibitor; IHD, ischemic heart disease; NIHD, non-ischemic heart disease; ECMO, extracorporeal membrane oxygenation; IABP, intra-aortic balloon pump.

**Table S4.** Culture data and treatment of HeartMate 3 patients that developed LSIs, by center.

| **Variable** | **Total (N = 71)** | **University of Pennsylvania (n = 15)** | **Mount Sinai Health Systems (n = 56)** | **P Value** |
| --- | --- | --- | --- | --- |
| **Microbiology** |  |  |  |  |
| **Culture Negative** | 4 (5.6%) | 1 (6.7%) | 3 (5.4%) | 1.000 |
| **Polymicrobial** | 5 (7.0%) | 2 (13.3%) | 3 (5.4%) | 0.368 |
| **Gram Positive** | 44 (62.0%) | 10 (66.7%) | 34 (60.7%) | 0.673 |
| ***Staphylococcus*** | 35 (49.3%) | 10 (66.7%) | 25 (44.6%) | 0.130 |
| **MSSA** | 23 (32.4%) | 6 (40.0%) | 17 (30.4%) | 0.541 |
| ***S. epidermidis*** | 8 (11.3%) | 1 (6.7%) | 7 (12.5%) | 0.460 |
| **MRSA** | 1 (1.4%) | 0 (0.0%) | 1 (1.7%) | 0.211 |
| ***S. schleiferi*** | 2 (2.8%) | 2 (13.3%) | 0 (0.0%) | 0.042 |
| ***Corynebacterium*** | 5 (7.0%) | 1 (6.7%) | 4 (7.1%) | 0.717 |
| ***C. jeikeium*** | 1 (1.4%) | 0 (0.0%) | 1 (1.7%) | 0.789 |
| ***C. striatum*** | 2 (2.8%) | 0 (0.0%) | 2 (3.6%) | 0.620 |
| ***Enterococcus faecalis*** | 2 (2.8%) | 0 (0.0%) | 2 (3.6%) | 0.620 |
| ***Streptococcus dysgalactiae*** | 1 (1.4%) | 0 (0.0%) | 1 (1.4%) | 0.789 |
| ***Granulicatella adiacens*** | 1 (1.4%) | 0 (0.0%) | 1 (1.4%) | 0.789 |
| **Gram Negative** | 28 (39.4%) | 4 (26.6%) | 21 (37.5%) | 0.550 |
| ***Pseudomonas*** | 11 (15.5%) | 3 (20.0%) | 8 (14.3%) | 0.689 |
| ***P. aeruginosa*** | 7 (9.9%) | 3 (20.0%) | 4 (7.1%) | 0.158 |
| ***P. fluorescens*** | 1 (1.4%) | 0 (0.0%) | 1 (1.7%) | 0.789 |
| ***Serratia*** | 6 (8.5%) | 0 (0.0%) | 6 (10.7%) | 0.227 |
| ***S. marcescens*** | 2 (2.8%) | 0 (0.0%) | 2 (3.6%) | 0.620 |
| ***Proteus*** | 5 (7.0%) | 0 (0.0%) | 5 (8.9%) | 0.293 |
| ***P. mirabilis*** | 4 (5.6%) | 0 (0.0%) | 4 (7.1%) | 0.378 |
| ***P. vulgaris*** | 1 (1.4%) | 0 (0.0%) | 1 (1.4%) | 0.789 |
| ***Enterobacter cloaecae*** | 2 (2.8%) | 1 (6.7%) | 1 (1.7%) | 0.380 |
| ***Stenotrophomonas maltophilia*** | 1 (1.4%) | 0 (0.0%) | 1 (1.4%) | 0.789 |
| ***Acinetobacter baumannii*** | 1 (1.4%) | 0 (0.0%) | 1 (1.7%) | 0.789 |
| **Fungal** | 1 (1.4%) | 1 (6.7%) | 0 (0.0%) | 0.211 |
| ***Candida albicans*** | 1 (1.4%) | 1 (6.7%) | 0 (0.0%) | 0.211 |
|  |  |  |  |  |
| **Treatment Course** |  |  |  |  |
| **Time to Infection, d** | 231 (112-423) | 208 (66-436) | 242 (114-416) | 0.597 |
| **Bloodstream Infection** | 15 (21.1%) | 3 (20.0%) | 12 (21.4%) | 1.000 |
| **Mediastinitis** | 9 (12.7%) | 1 (6.7%) | 8 (14.3%) | 0.673 |
| **Pocket Infection** | 12 (16.9%) | 3 (20.0%) | 9 (16.1%) | 0.708 |
| **Length of Antimicrobial Therapy, d** | 361 (141-604) | 195 (69-601) | 418 (202-656) | 0.096 |
| **Required Suppressive Antimicrobial Therapy** | 55 (77.5%) | 10 (66.67%) | 45 (80.4%) | 0.303 |
| **Required Surgical Reintervention** | 20 (28.2%) | 3 (20.0%) | 17 (30.4%) | 0.531 |
| **Surgical Driveline Debridement** | 19 (26.8%) | 3 (20.0%) | 16 (28.6%) | 0.744 |
| **Device Exchange** | 3 (4.2%) | 0 (0.0%) | 3 (5.4%) | 0.485 |
| **Required Hospitalization for LSI** | 53 (74.7%) | 14 (93.3%) | 39 (69.6%) | 0.061 |
| **Number of LSI-Related Hospitalizations** | 1 (0-2) | 2 (1-3) | 1 (0-2) | 0.181 |
| **Days Hospitalized for LSI** | 8 (0-28) | 10 (6-28) | 7 (0-27) | 0.464 |

Categorical data are expressed as n (%) and continuous data as medians (interquartile range). Bold type denotes P < 0.05. LSI, LVAD-specific infection; MSSA, methicillin-sensitive *Staphylococcus aureus*; MRSA, methicillin-resistant *S. aureus.*
